# The Genetic Research in Alzheimer Disease (GERALD) Initiative Finds rs9320913 as a Neural eQTL of lincRNA AL589740.1

**DOI:** 10.1155/2021/3064224

**Published:** 2021-09-03

**Authors:** Lidia Lopez-Gutierrez, José María García-Alberca, Silvia Mendoza, Esther Gris, María Paz De la Guía, José Manuel Marin-Carmona, Emilio Alarcón-Martín, Almudena Lobato, Jose Manuel Cruz-Gamero, Laura Cura, Olga Ocejo, Javier Torrecilla, María Dolores Nieto, Concepción Urbano, Nuria Pareja, Macarena Luque, María García-Peralta, Rosario Carrillejo, José Luis Royo

**Affiliations:** ^1^Departamento de Especialidades Quirúrgicas, Bioquímica e Inmunología, Facultad de Medicina, Universidad de Málaga, Blv. Luis Pasteur s/n. 29071 Málaga, Spain; ^2^Instituto Andaluz de Neurociencia (IANEC), Calle Álamos, 17, 29012 Málaga, Spain; ^3^Asociación de Familiares de Personas con Alzheimer, Málaga, Camino de los Almendrales, 35, 29013 Málaga, Spain; ^4^Centro Residencial Almudena, Calle Galicia, 3, 29730 Rincón de la Victoria, Spain; ^5^Hospital Hermanas Hospitalarias del Sagrado Corazón, Calle San Juan Bosco, 41, 29014 Málaga, Spain; ^6^Asociación de Familiares de Enfermos de Alzheimer de la Axarquía, Calle Francisco Labao Gámez, 29700 Vélez-Málaga, Spain; ^7^Centro Residencial Élite, Calle Picos de Europa, 23, 29018 Málaga, Spain; ^8^Asociación Criptana de Enfermos de Alzheimer, Calle Álvarez de Castro, s/n. 13610 Campo de Criptana, Spain

## Abstract

Alzheimer's disease is the most common cause of dementia worldwide, and longitudinal studies are crucial to find the factors affecting disease development. Here, we describe a novel initiative from southern Spain designed to contribute in the identification of the genetic component of the cognitive decline of Alzheimer's disease patients. The germline variant rs9320913 is a C>A substitution mapping within a gene desert. Although it has been previously associated to a higher educational achievement and increased fluid intelligence, its role on Alzheimer's disease risk and progression remains elusive. A total of 407 subjects were included in the study, comprising 153 Alzheimer disease patients and 254 healthy controls. We have explored the rs9320913 contribution to both Alzheimer disease risk and progression according to the Mini-Mental State Exams. We found that rs9320913 maps within a central nervous system lincRNA AL589740.1. eQTL results show that rs9320913 correlated with the brain-frontal cortex (beta = −0.15, *p* value = 0.057) and brain-spinal cord (beta of -0.23, *p* value = 0.037). We did not find rs9320913 to be associated to AD risk, although AA patients seemed to exhibit a less pronounced Mini-Mental State Exam score decline.

## 1. Introduction

Alzheimer's disease (AD) diagnosis involves a significant decline of intellectual abilities in one or more cognitive domains including learning and memory, language, and executive functions [1, 2]. A study conducted by Stern et al. [3] indicated that individuals with a low level of schooling had an approximately twofold increased risk of developing dementia pinpointing the protective role of cognitive reserve on AD risk. Moreover, a higher cognitive reserve is associated with a significant reduction in the risk of symptom onset of mild cognitive impairment, which appears during the prodromal stage of AD. This delay in the onset of cognitive problems can be up to seven years [4]. Thus, previous genetic correlations suggest protective effects of the cognitive reserve for Alzheimer's disease [5]. Different elements are associated with a greater cognitive reserve including the educational level and occupational and physical activities [6, 7]. Intelligence is a highly heritable trait and a net contributor to individual cognitive reserve [8, 9]. Recently, a Genome-Wide Association analysis comprising 78,308 subjects linked rs9320913 with fluid intelligence [10]. A previous work also associated rs9320913-A allele with a higher educational level and academic success [11]. The initial characterization of rs9320913 revealed that it lies within its own linkage disequilibrium block in a 1.6 Mb gene desert, 859 kb and 700 kb away of the nearest protein-coding genes. Further analysis of the genetic region showed that the polymorphism was located within AL589740.1 (ENCODE reference ENSG00000271860), a long intervening noncoding RNA (lincRNA) whose longer transcript spans along 570 kb and 16 exons with 51 mature splice variants ranging from 0.3 to 3 kb. Unfortunately, their physiological role, as for many lincRNAs, is still unknown. This fact brings special relevance to the study of rs9320913, since lincRNAs have been associated to the molecular etiology of several neurodegenerative disorders including AD ([12] and references therein). In this context, we launched the Genetic Research in Alzheimer Disease (GERALD) initiative, a retrospective and prospective longitudinal project aimed at recruiting AD patients from Málaga (Southern Spain). The present work shows the pipeline of the project and the first results, derived from studying the rs9320913 variant in the course of the AD.

## 2. Materials and Methods

### 2.1. Study Population

**Figure 1:**
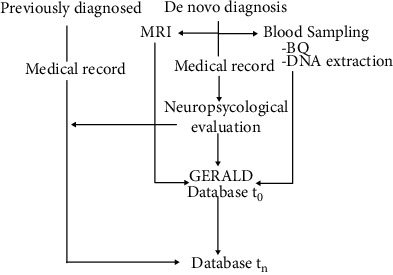
Diagram showing GERALD project pipeline. De novo diagnoses undergo a full neuropsychological evaluation together with CNS magnetic resonance imaging (MRI). Peripheral blood extraction is used for both biochemical (BQ) analysis and DNA extraction for ApoE genotyping. Relevant data from the medical records, together with the different tests results, are integrated in a database. Subsequent follow-up results are also digitalized. Previously diagnosed patients undergo the neuropsychological evaluation which is longitudinally revised.

The Genetic Research in Alzheimer Disease initiative is led by the Instituto Andaluz de Neurociencia, Málaga (https://ianec.es/), from Spain.  Figure 1 summarizes the recruitment pipeline. Additional recruiting centers include the Asociación de Familiares de Alzheimer, Málaga; the Hospital Hermanas Hospitalarias del Sagrado Corazón, Málaga; the Centro Residencial Almudena, Rincón de la Victoria; the Centro Residencial Élite, Málaga; the Asociación de Familiares de Enfermos de Alzheimer dela Axarquía; and the Asociación Criptana de Enfermos de Alzheimer, Campo de Criptana, all of them in Spain. Inclusion criteria were the diagnosis of late onset Alzheimer disease according to the benchmarks set forth by the National Institute on Aging Alzheimer's Association (NIA/AA) workgroups [12]. Patients were Caucasian with Mediterranean origin. All of them were followed during at least 6 months and provided either a blood sample or buccal swab that was used for DNA extraction. A single code was used for patient anonymization. Whenever required, brain magnetic resonance imaging was acquired on a 1.5 or 3.0 Tesla General Electric Signal scanner and rated by a neurologist, blinded to the genetic and clinical data. White matter hyperintensities were visually rated from a 0 (no hyperintensities) to 6 (severe and profuse hyperintensities). The relevant data from the medical records were digitalized. The present manuscript comprised 153 AD patients, although the recruitment is open. In addition, 254 healthy students of the School of Medicine from the University of Málaga enrolled in the project as population controls. Inclusion criteria were being adult and feeling healthy without reported psychiatric disease. This research was carried out with the approval of the Ethics Committee of the University of Málaga. Subjects included in the series were recruited between February 2017 and June 2019. The study strictly follows the ethical standards adopted by the XVIII World Medical Assembly Declaration of Helsinki and subsequent revisions.

### 2.2. DNA Extraction and Genotyping

DNA was extracted from each donor buccal swap using conventional digestion followed by ethanol precipitation. Blood DNA was extracted with a Blood Genomic DNA Extraction Kit (Canvax SL, Córdoba, Spain) following the manufacturer's instructions. SNP rs9320913 was genotyped by PCR and subsequent enzymatic digestion using the following primers: forward 5′-TCACATTTTCCTGCATACCG-3′ and reverse 5′-AAAACCTGGATAAAGCAATCAA-3′. Products were amplified in an Applied Biosystems 2700 thermocycler, using 30 cycles of 94°C for 30s, 60°C for 30s, and 72°C for 30s. Amplicons sizing 242 bp were digested with Apo I (Thermo Fisher Scientific, Massachusetts, USA) at 50°C for 1.5 hour and further run in a 3% agarose gel. The presence of the rs9320913-A allele generated two fragments of 172 and 70 bp, while the rs9320913-C allele retained the original length (Supplementary Figure 1). For the determination of the APOE genotype, the region comprising rs429358 and rs7412 was amplified by PCR with the oligonucleotides 5′-ACGGCTGTCCAAGGAGCTGC-3′ and 5′-CGGCTGCCATCTCCTCCATCC-3′. PCRs were performed at a 20 *μ*l volume using 10 pg of each primer and 1x TB premix Ex (Takara Bio Inc., Japan) with 5% dimethyl sulfoxide (DMSO), in an Applied Biosystems 2700 thermocycler with the following conditions: 95°C for 5 min followed by 35 cycles of 94°C for 30 sec, 65°C for 30 sec, and 72°C for 30 sec. Next, PCRs were split in two independent LightCycler® Capillaries, each of them with 9 *μ*l of the PCR product and 2 pg of the corresponding probe mix (rs429358 Anchor: 5′-CTGCAGGCGGCGCAGGCCCGGCTGGGCGC[fluorescein]-3′ and sensor: 5′-[Cy5]ACATGGAGGACGTGCGCGG[phosphate]-3′; rs7412 Anchor: 5′-GCTGCGTAAGCGGCTCCTCCGCGATGCCG[fluorescein]-3′ and sensor: 5′-[Cy5]GACCTGCAGAAGCGCCTGGC[phosphate]-3′). The fluorescence measurement was monitored using a LightCycler® (Roche Applied Science, Germany). For rs429358 (codon 112) determination, allele C exhibited a melting temperature (Tm) of 63° C, in contrast to the Tm = 55°C displayed by allele T. For rs7412 (codon 176), the C allele exhibited a Tm of 65° C while allele T had a Tm = 58°C (Supplementary Figure 2).

### 2.3. Statistical Analysis

The Hardy–Weinberg equilibrium was assayed using the corresponding online web tool from the Institute of Human Genetics of Munich (https://ihg.gsf.de/ihg/snps.html). Statistical analysis was performed using IBM SPSS Statistics v22. Power studies were calculated with Rothman's Episheet [13]. The Kolmogorov–Smirnov test was used to determine the normality of the quantitative data series. For bivariate correlation studies, Pearson's correlation coefficient and Spearman's Rho were calculated. In order to evaluate cognitive decline, the slope between two Mini Mental State Examination (MMSE) scores obtained at AD diagnosis and at the first follow-up was obtained. Only data from the Q2 to Q4 quartiles of the MMSE scores at the time of diagnosis were taken into account for a better assessment of cognitive decline. The level of significance was defined at 0.05.

## 3. Results

### 3.1. GERALD Series

Subjects comprised 121 women and 39 men, whose age at the time of diagnosis was from 39 to 91 years (mean age 77.21 ± 7.97 years), of which 76.7% (82.0% women and 18.0% men) presented a low educational level. The mean duration of symptoms determined through interviews with caregivers was 45.22 ± 31.79 months. MMSE scores determined at recruitment were 19.5 ± 5.9 points (average ± standard deviation). Global deterioration scores at recruitment ranged between 0.5 and 3, with an average of 1.98. Cognitive decline measured with MMSE varied between 0.78 and -2.22 points/month. When ApoE genotypes were determined, a single E4 allele was found in 55 (36.2%) patients, while homozygosity was found in 14 (9.2%). Deep white matter intensities ranged between 0 and 24, with an average of 5.1. Hippocampal atrophy ranged between 0 and 8, with an average of 3.37.

### 3.2. Study of rs9320913 in AD Etiology

**Figure 2:**
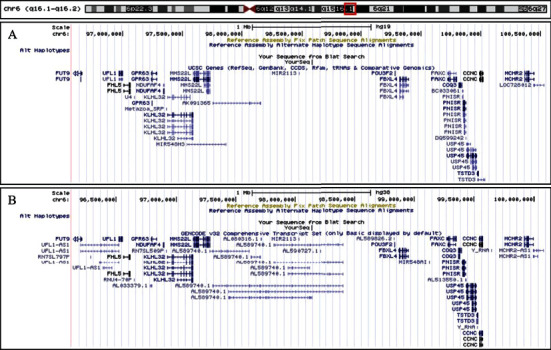
Region of polymorphism rs9320913 (Chr6: 98136857) searched on hg19 (a) and hg38 (b) genome browser versions.

**Figure 3:**
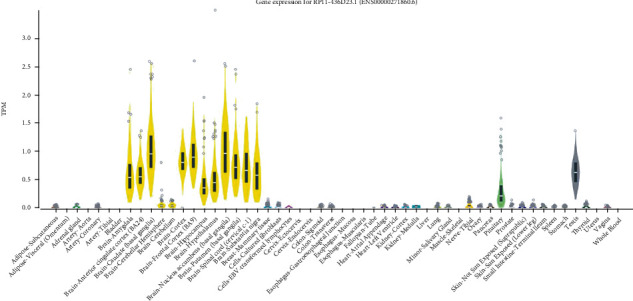
Violin plots representing AL589740.1 gene expression measured in transcripts per kilobase million (TPM) according to the GTEX portal (https://gtexportal.org). Significant expression is detected in different central nervous system areas, pituitary, and testis.

First reports mapped rs9320913 on a genetic desert on chromosome 6 flanked by POU3F2 and MMS22L, with no evident candidate gene potentially associated to the intelligence phenotype (Figure 2(a)). Our search was realized using the updated hg38 version of the genome browser, which revealed that rs9320913 now maps a long intervening noncoding RNA referenced AL589740.1 (Figure 2(b)). Moreover, when the AL589740.1 expression profile was explored in the GTEx portal, we found that its expression patterns were mainly constrained to the central nervous system (CNS) (Figure 3). Next, we examined whether rs9320913 genotype may be associated to the expression levels of AL589740.1 (expression quantitative trait locus or eQTL). Results showed that rs9320913 correlated with the brain-spinal cord (*β* = −0.23, *p* value = 0.037) and showed a trend towards association with the brain-frontal cortex (BA9) (*β* = -0.15, *p* value = 0.057) (Supplementary Table 1). In order to check whether the effect of rs9320913 might be extended to the closest protein-coding genes, we analyzed whether these genes might also exhibit a CNS-centered pattern. While in the centromeric border, MMS22L showed a virtually ubiquitous expression pattern; in the telomeric side, POU3F2 showed a CNS-associated expression pattern (Supplementary Figure 3). Although eQTL analysis for POU3F2 did not show any particular trend towards association, results for MMS22L indicate a correlation between rs9320913 and brain-amygdala gene expression (beta = 0.15, *p* value = 0.042) (Supplementary Table 1). These results suggest that rs9320913 acts as eQTL for AL589740.1 and could also act on MMS22L, but in different regions of the CNS.

When rs9320913 was genotyped in both case and control series, we observed that distributions fit the Hardy–Weinberg equilibrium. Allele frequencies were slightly different between cases and controls, since AA individuals were underrepresented in the group of cases: 15% versus the 21.2% found among the control group (Table 1). However, these results did not reach statistical difference. When the study power was post hoc checked, we found that we were 80% powered to detect odds ratios ≥ 0.47. This suggests that the effect size of rs9320913 in AD risk is, if any, below OR = 0.47.

When the role of rs9320913 was examined according to the MMSE at diagnostics, no significant differences were observed between genotypes. The educational level and the score obtained in the MMSE test at the time of diagnosis showed a positive correlation (Spearman′s rho correlation = 0.308, *p* value < 0.001) (Supplementary Figure 4). The age of onset showed correlation with the MMSE test score at the time of diagnosis (Spearman's rho correlation of -0.405, *p* value < 0.001) as well as an association with the educational level (Spearman's rho correlation of -0.189, *p* value = 0.017). The inverse association between age of onset and the MMSE score obtained indicates a trend towards lower MMSE scores as patient age increases (*β* = −0.345, *p* value < 0.001), while the effect of educational level was proven to be positive, as higher educational levels are related to higher MMSE scores at diagnosis (*β* = 0.255, *p* value = 0.001). No significant associations were found between any of the clinical variables measured and the rs9320913 genotype. Thus, the previously reported effect of this variant on the educational level could not be confirmed.

**Figure 4:**
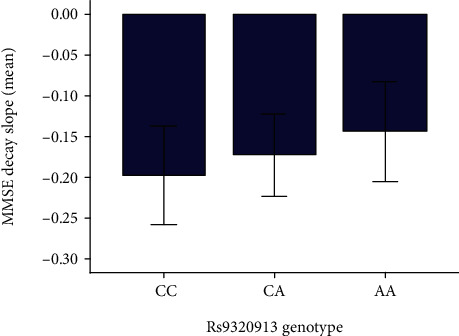
Bar chart of distribution of mean values of slope according to polymorphism genotype.

Regarding the patient's cognitive decline, this was measured by consecutive MMSE tests, generating the variable named MMSE decay slope of the first follow-up. Equivalently, these calculations were performed for GDS. As expected, both parameters were negatively correlated (beta = −0.18, *p* value = 0.028, Pearson's test). Given its higher dynamic range, only MMSE decay was included in further analysis. Interestingly, MMSE decay showed a trend towards less cognitive decline in homozygous rs9320913-A individuals, although results were not statistically significant probably due to the limited sample size.  Figure 4 presents the mean values of slope according to the rs9320913 genotype, which corresponds to −0.198 ± 0.444 points per month for the CC genotype, −0.173 ± 0.355 points per month for CA patients, and −0.144 ± 0.262 points per month for those patients with the AA genotype. We observed that individuals with A-allele tend to show a higher educational level. Linear regression carried out taking MMSE decay is shown in Table 2. No correlation was found between the MMSE decay and any of the considered variables. Although several studies point to a possible effect of this variant in the development of Alzheimer's, given its involvement in intelligence, in the present work, only a negative trend was found between cognitive decline and academic success.

## 4. Discussion

Here, we present the creation of a novel longitudinal Alzheimer disease cohort devoted to contribute to revealing new genetic factors affecting AD cognitive decline. In this first work, we have focused on the characterization of the polymorphism rs9320913 given its potential role in cognitive reserve. Previous studies have reported the association between genetic variants and educational attainment and intelligence as well as an inverse relationship with the appearance of Alzheimer's disease and depressive symptoms [10, 11, 14]. A protective effect between intelligence and disease development has been proposed [14]. As rs9320913 has been related to academic success [10, 11], our starting hypothesis was that the variant, being involved in processes that contribute to cognitive reserve, might influence the development of the disease. Thus, the effect of rs9320913 on the progression of AD is measured in cognitive decline using serial MMSE examinations. After genotyping the patient and control samples and performing the corresponding Hardy–Weinberg balance study, a statistical study was carried out in search of the relationships that could be established between the genotype of the variant and the cognitive decline due to AD.

Reviewing the genomic landscape of rs9320913, we found that it is located in a lincRNA, AL589740.1, of unknown function, but with a high expression in the central nervous system. This may suggest roles in neurodevelopmental and other processes specific to the central nervous system. In this regard, several lncRNAs have been linked to regulation of neuronal apoptosis, providing a new perspective on neurodegeneration [15]. lincRNA MALAT1, for instance, has been shown to inhibit neuron apoptosis and neuroimflammation [16], and lincRNA H19 has been found to initiate microglial pyroptosis [17].

The polymorphism rs9320913 turned out to be eQTL from AL589740.1, with significant association in the brain-spinal cord and brain-frontal cortex (BA9). A negative relation between the variant and the expression level was revealed. The implication of a lincRNA was of special interest as it has been reported that these molecules are expressed aberrantly during neurodegeneration, although their involvement in the process remains unclear. However, their possible effect on gene expression could establish a greater difference between patients and controls [18]. Nevertheless, a correlation between the rs9320913 genotype and the MMS22L expression has also been stablished, so we cannot discard a possible effect of the variant on an enhancer involved in the control of the MMS22L expression. However, the ubiquitous expression pattern of this gene diminishes the possible importance that its alteration can have on the development of intelligence.

Previous studies show the relationship between this allele and greater intelligence [10, 11]. Based on the theory of cognitive reserve, which argues that this ability optimizes cognitive functioning, it is expected that any effect on this capacity would affect the progression of the cognitive impairment associated with the disease. Despite this, no effect was found either on the parameter measuring decline or on the age of onset of cognitive impairment. Both the MMSE test score and age of onset were related to the educational level. Individuals with a higher educational level obtain higher scores in the MMSE test at the time of diagnosis, as was expected in the basis of the cognitive reserve theory [4]. However, results for age of onset were conflicting, as this variable showed a negative correlation with the educational level and this would imply that people with a higher cognitive reserve would present the symptoms earlier than those with a low cognitive reserve. An alternative explanation for this result is the greater sensitivity of highly educated people to their cognitive care, so that these people would detect symptoms earlier, while those with lower educational levels would contact a professional with a greater deterioration. On the other side, specifically in the cohort of patients studied, the scale of the educational level used must be taken into account. The category of high educational level corresponds to patients with a bachelor's degree and higher education levels, so there will be no differentiation among university levels. Other researches evaluate the effect of education based on years of schooling, which is a more precise scale to discern the effect of education on the development of the disease through cognitive reserve.

The statistical study carried out with rs9320913 did not reveal any significant association. However, the cognitive decline tended to be less pronounced in the presence of the A-allele, which would mean that this variant is associated to a less severe fall. Concerning the educational attainment, a trend towards a higher educational level was found related to the A-allele, which could be pointing out the effect of this variant on intelligence development. Both variables, MMSE decay slope and educational level, were found to be inversely related, and from this, we could extract either an effect of the polymorphism on the educational level, which in turn affects the lower cognitive decline, or a direct effect of the variant on both parameters. Nevertheless, the lack of statistical power of the study makes it impossible to validate these results and extract a clear conclusion. An extension of the sample size would be convenient to verify the results obtained, as well as the application of the parameter over a wider range.

Besides, we have focused on the MMSE test as a proxy for cognitive impairment, as it is the most widely used given its rapid clinical application and the proper information it offers in patients with typical Alzheimer's disease [19].

## Figures and Tables

**Table 1:** rs9320913 genotypes in the case and control series.

	Controls (*n* = 254)	Cases (*n* = 153)	OR	*p* value
CC	88	61	—	
CA	112	69	0.89	0.60^a^
AA	54	23	0.61	0.10^b^
HWE *p* value	0.10	0.63		

^a^Dominant model (CC vs. GA); ^b^recessive model (C- vs. AA), according to the Munich University web tool.

**Table 2:** Lineal regression adjustment model taking the *slope* variable dependent on educational level, age of onset, ApoE genotype, and rs9320913 genotype.

	MMSE decay	
	Beta	*p* value
Educational level	0.067	0.531
Age of onset	-0.011	0.919
APOE genotype	-0.190	0.074
rs9320913 genotype	0.028	0.790

Slope: ANOVA analysis *F* value = 1.055 (*p* value 0.384).

## Data Availability

Bioinformatic analysis is explained in the text and therefore can be independently reproduced. Raw personal data of Alzheimer patients is available upon the favorable pronunciation of the regional ethics committee.

## Supplementary Materials

Supplementary Materials Supplementary Figure 1: rs9320913 restriction fragment length polymorphism using Apo I. Supplementary Figure 2: ApoE genotyping plots obtained from a double heterozygote. Panel A shows the results for rs429358 while panel B refers to rs7412. Supplementary Figure 3: violin plots representing MMS22L (A) and POU3F2 (B) gene expression measured in transcripts per kilobase million (TPM) according to GTEX portal (https://gtexportal.org). MMS22L shows ubiquitous expression while POU3F2 present significant expression is detected in different central nervous system areas and the nerve. Supplementary Figure 4: boxplot of distribution of MMSE scores at the time of diagnosis according to educational level. Supplementary Table 1: eQTL between rs9320913 and AL589740.1, POU3F2, and MMS22l in the different tissues of the central nervous system.
