# Supplementary material for: The Genetic Research in Alzheimer Disease (GERALD) Initiative Finds rs9320913 as a Neural eQTL of lincRNA AL589740.1
Source: Int J Alzheimers Dis. 2021 Sep 3;2021:3064224. doi: 10.1155/2021/3064224 (PMC8455222; doi:10.1155/2021/3064224)
Supplement: Supplementary Materials — Supplementary Figure 1: rs9320913 restriction fragment length polymorphism using Apo I. Supplementary Figure 2: ApoE genotyping plots obtained from a double heterozygote. Panel A shows the results for rs429358 while panel B refers to rs7412. Supplementary Figure 3: violin plots representing MMS22L (A) and POU3F2 (B) gene expression measured in transcripts per kilobase million (TPM) according to GTEX portal (https://gtexportal.org). MMS22L shows ubiquitous expression while POU3F2 present significant expression is detected in different central nervous system areas and the nerve. Supplementary Figure 4: boxplot of distribution of MMSE scores at the time of diagnosis according to educational level. Supplementary Table 1: eQTL between rs9320913 and AL589740.1, POU3F2, and MMS22l in the different tissues of the central nervous system. [file 3064224.f1.zip › Supplementary table 1.docx]

**Supplementary Table**

**Supplementary table 1.** eQTL between rs9320913 and AL589740.1, POU3F2 and MMS22l in the different tissues of the central nervous system.

| **Tissue** | **AL589740.1** | | **POU3F2** | | **MMS22L** | |
| --- | --- | --- | --- | --- | --- | --- |
|  | **Beta** | **p-value** | **Beta** | **p-value** | **Beta** | **p-value** |
| Brain- Spinal cord (cervical c-1) | -0.23 | **0.037*** | -0.064 | 0.39 | -0.0360 | 0.70 |
| Brain- Frontal Cortex (BA9) | -0.15 | **0.057*** | 0.0056 | 0.95 | -0.092 | 0.33 |
| Brain- Hypothalamus | 0.21 | 0.12 | 0.1 | 0.31 | 0.068 | 0.41 |
| Brain- Cortex | -0.12 | 0.14 | -0.078 | 0.14 | 0.059 | 0.43 |
| Brain- Amygdala | -0.16 | 0.15 | -0.019 | 0.82 | 0.15 | **0.042*** |
| Brain- Anterior cingulate cortex (BA24) | -0.12 | 0.2 | 0.053 | 0.42 | -0.018 | 0.83 |
| Brain- Substantia nigra | 0.15 | 0.21 | -0.057 | 0.48 | 0.16 | 0.17 |
| Brain- Caudate (basal ganglia) | 0.11 | 0.31 | 0.021 | 0.67 | 0.057 | 0.44 |
| Brain – Putamen (basal ganglia) | -0.11 | 0.35 | -0.033 | 0.58 | 0.015 | 0.89 |
| Brain – Nucleus accumbens (basal ganglia) | 0.024 | 0.8 | -0.086 | 0.21 | -0.15 | 0.061 |
| Brain – Hippocampus | 0.024 | 0.82 | -0.058 | 0.36 | -0.06 | 0.36 |
