# Supplementary figures and images for: The Genetic Research in Alzheimer Disease (GERALD) Initiative Finds rs9320913 as a Neural eQTL of lincRNA AL589740.1
Source: Int J Alzheimers Dis. 2021 Sep 3;2021:3064224. doi: 10.1155/2021/3064224 (PMC8455222; doi:10.1155/2021/3064224)

**Supplementary Figure 1.**

**
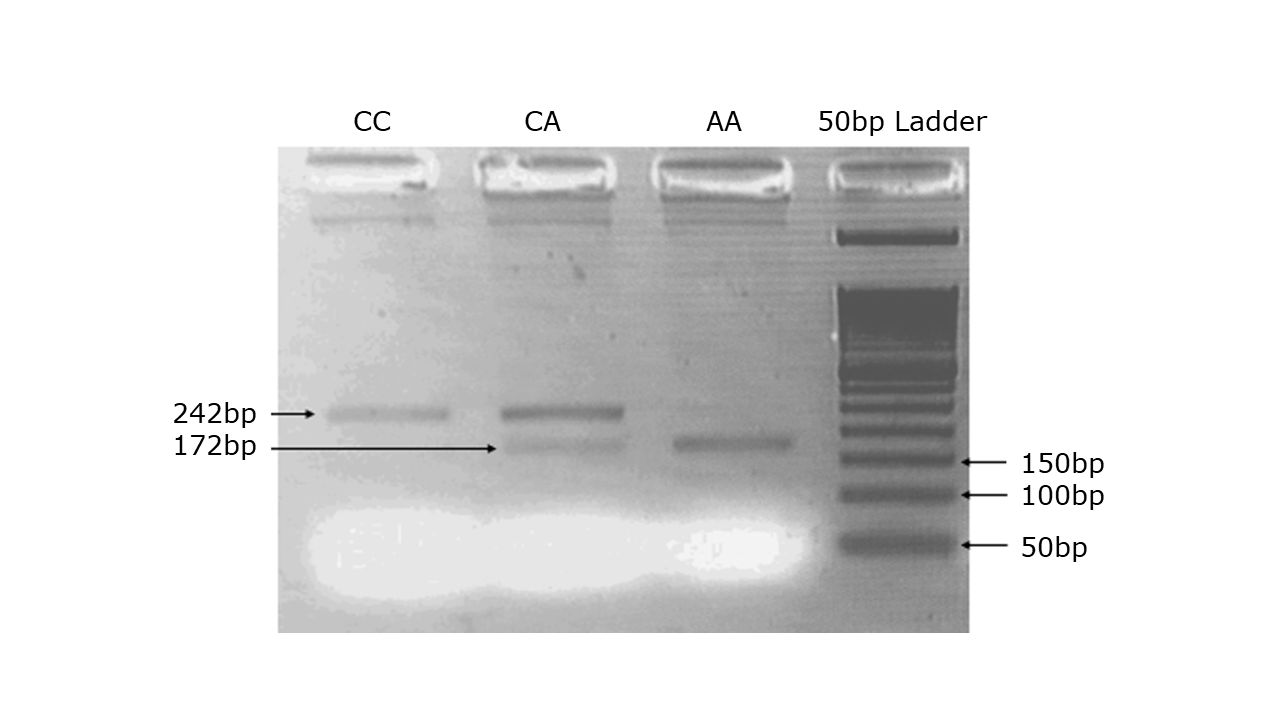
**

**Supplementary Figure 2.**

**
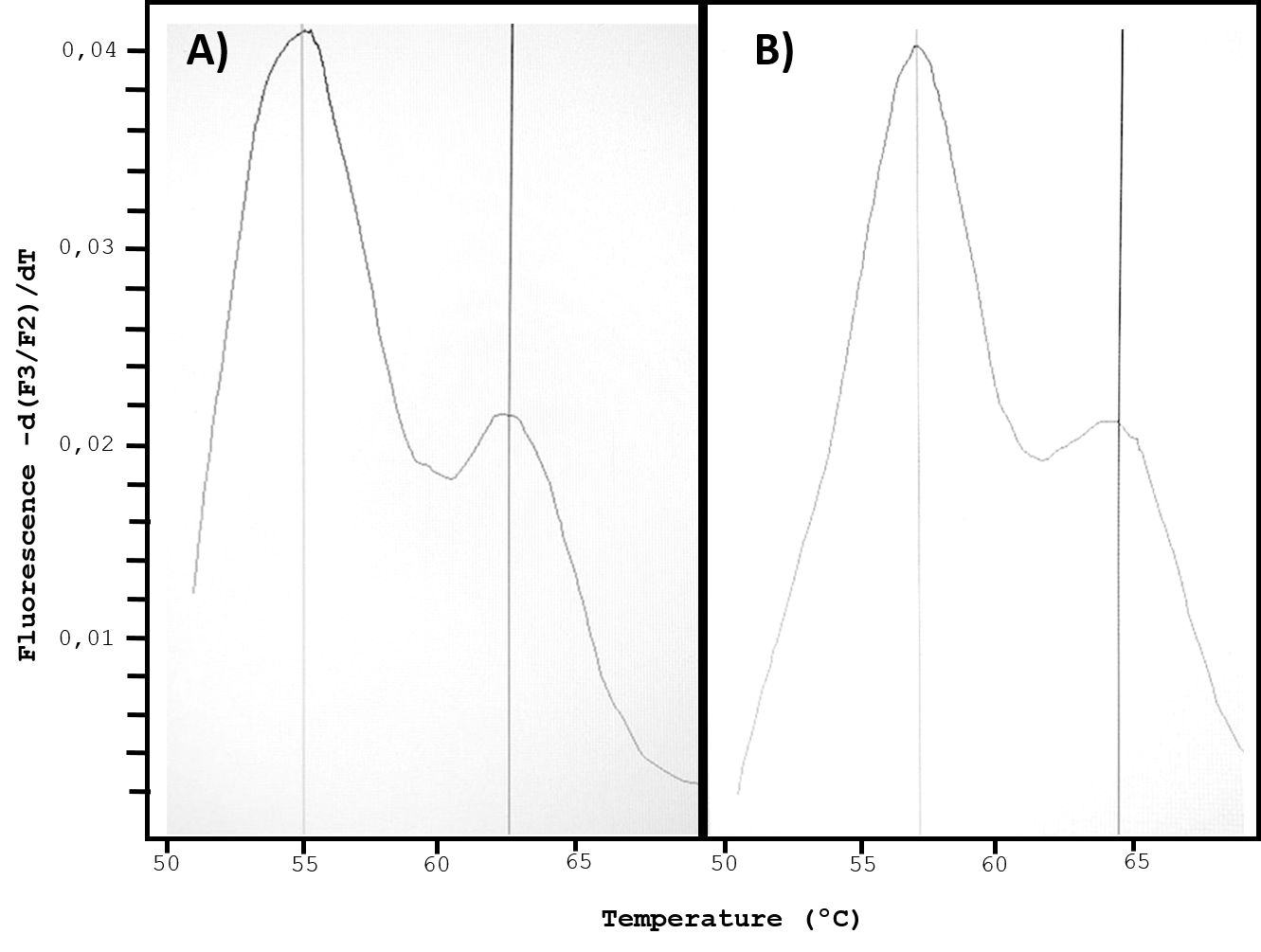
**

**Supplementary Figure 3.**


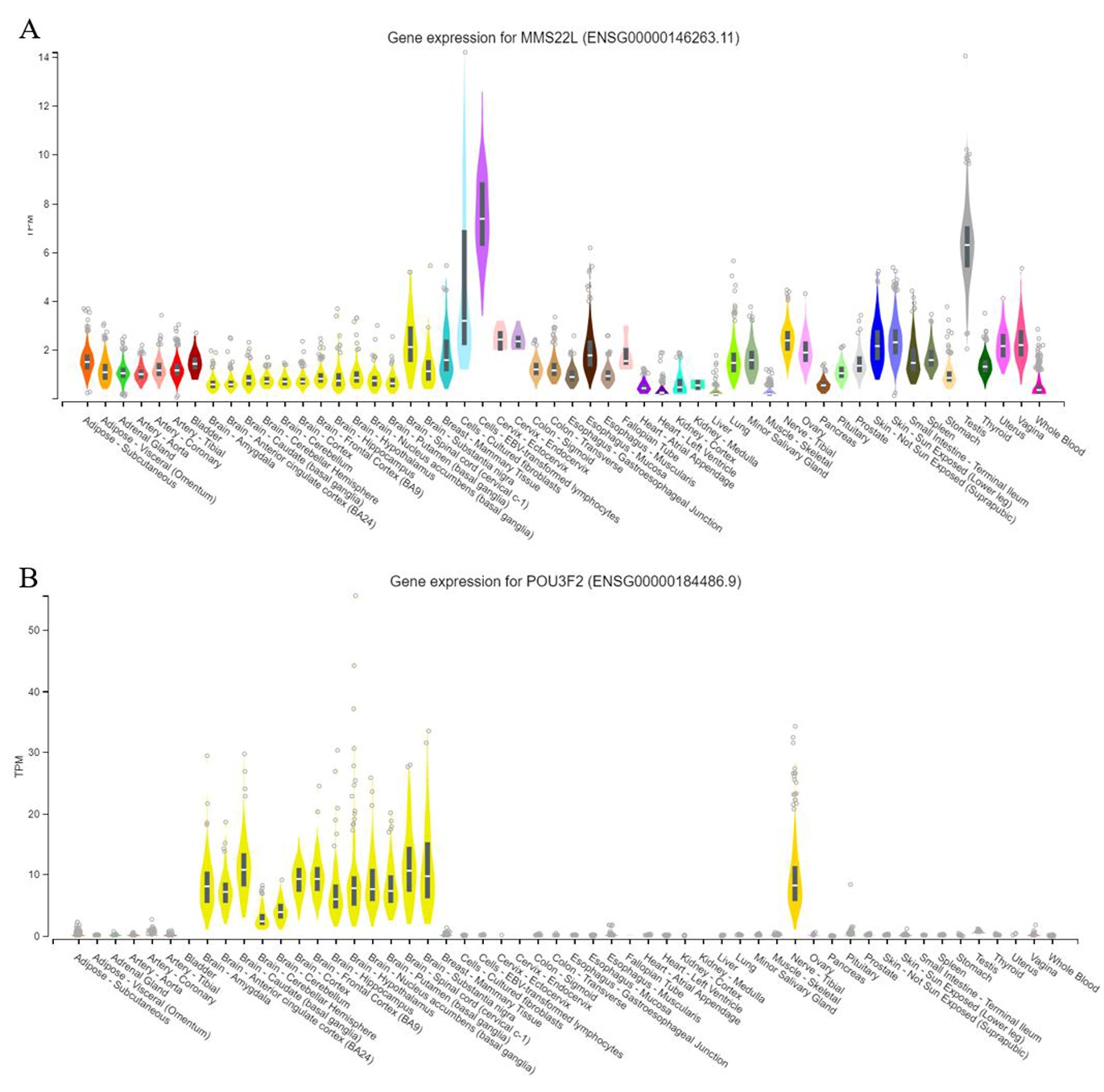


**Supplementary Figure 4.**


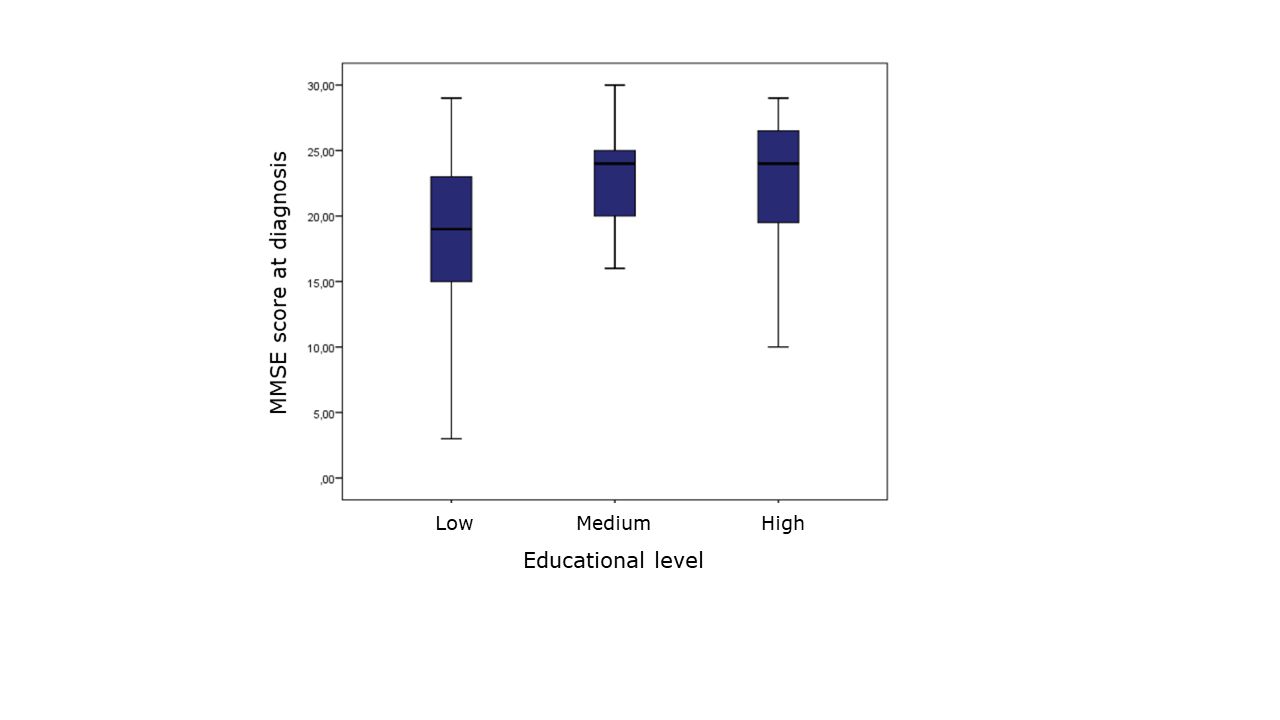

Supplement: Supplementary Materials — Supplementary Figure 1: rs9320913 restriction fragment length polymorphism using Apo I. Supplementary Figure 2: ApoE genotyping plots obtained from a double heterozygote. Panel A shows the results for rs429358 while panel B refers to rs7412. Supplementary Figure 3: violin plots representing MMS22L (A) and POU3F2 (B) gene expression measured in transcripts per kilobase million (TPM) according to GTEX portal (https://gtexportal.org). MMS22L shows ubiquitous expression while POU3F2 present significant expression is detected in different central nervous system areas and the nerve. Supplementary Figure 4: boxplot of distribution of MMSE scores at the time of diagnosis according to educational level. Supplementary Table 1: eQTL between rs9320913 and AL589740.1, POU3F2, and MMS22l in the different tissues of the central nervous system. [file 3064224.f1.zip › Supplementary Figures.docx]
